# Effect of Operational Conditions on the Behaviour and Associated Costs of Mixed Microbial Cultures for PHA Production

**DOI:** 10.3390/polym11020191

**Published:** 2019-01-22

**Authors:** Francisco Cabrera, Álvaro Torres, José Luis Campos, David Jeison

**Affiliations:** 1 Department of Chemical Engineering, Universidad de La Frontera, Av. Francisco Salazar 01145, Temuco 4780000, Chile; f.cabrera01@ufromail.cl (F.C.); alvaro.torres@ufrontera.cl (Á.T.); 2 Facultad de Ingeniería y Ciencias, Universidad Adolfo Ibáñez, Avda. Padre Hurtado 750, Viña del Mar 2520000, Chile; jluis.campos@uai.cl; 3 Escuela de Ingeniería Bioquímica, Pontificia Universidad Católica de Valparaíso, Av. Brasil 2085, Valparaíso 2362803, Chile

**Keywords:** PHA, mixed microbial cultures, bioplastics, feast-famine, cost

## Abstract

Massive production and disposal of petrochemical derived plastics represent relevant environmental problems. Polyhydroxyalkanoates (PHA) are a renewable alternative that can even be produced from wastes. The production of PHA from acetate using mixed microbial cultures was studied. The effect of two key operational conditions was evaluated, i.e., substrate concentration and cycle length. The effects of these factors on several responses were studied using a surface response methodology. Several reactors were operated under selected conditions for at least 10 solids retention times to ensure stable operation. Results show that conditions providing higher PHA content involve lower biomass productivities. This has a great impact on biomass production costs. Results suggest then that PHA content alone may not be a reasonable criterion for determining optimal conditions for PHB production. If production costs need to be reduced, conditions that provide a lower PHA content in the selection reactor, but a higher biomass productivity may be of interest.

## 1. Introduction

Plastics represent a serious environmental problem. They are usually non-biodegradable, are produced from non-renewable resources, and have low densities, meaning that they occupy a large volume in municipal landfills. Moreover, marine plastics pollution is a growing source of concern. It is mainly caused by single use plastics, which is rapidly changing policies and legislation in many countries around the world [1]. Polyhydroxyalkanoates (PHA) have been proposed as a potential replacement for traditional petrochemical based plastics, since they can be used in a wide range of industrial applications [2]. PHA are polyoxoesters of hydroxyalkanoic acids, which are synthesized by some bacteria as intracellular storage compounds [3]. They are biodegradable and can be produced from renewable resources [4].

The production of PHAs has been shown to be technically feasible when using known high-PHA accumulating bacterial cultures, like *Cupravidus necator* [5,6], or modified bacteria, like *Escherichia coli.* These microorganisms have reached internal PHA contents up to 90% dry weight when working with batch reactors [7]. The costs associated with inoculum preservation, raw materials, and downstream processing in PHA production make these polymers nowadays five to 10-fold more expensive than their fuel-based counterparts [8]. Moreover, when well defined substrates are used, this item can contribute one third of the operational costs [9]. In order to decrease the costs of inoculum preservation in axenic conditions, reduce the costs of raw materials, and increase the production efficiencies of PHAs, much research has been conducted during the last 20 years in the use of mixed microbial cultures (MMCs). The application of MMCs enables the use of volatile fatty acids (VFA) as substrates, which in turn can be derived from organic wastes by dark fermentation, further reducing potential costs and revalorizing waste organic compounds [10]. 

PHAs production using MMCs requires a first stage of culture enrichment of PHA accumulating microorganisms [11]. Transient conditions of carbon supply are normally used for this purpose: Consecutive phases of carbon source availability (feast) and scarcity (famine), which induces a selective pressure favouring the development of PHA accumulating microorganisms. This feast-famine operation strategy has also been regarded as aerobic dynamic feeding (ADF) [12]. Starvation for a certain period can cause a decrease in the amount of intracellular components needed for growth. After starvation, when the substrate is available again, storage occurs instead of growth since the amount of enzymes required for storage are lower than RNA and enzymes required for growth [13]. 

Several parameters have been identified as being relevant for the selection of MMC with an improved PHA accumulation capacity. Some of them are the organic load rate (OLR), influent substrate concentration, type of substrate, sludge retention time, temperature, pH, oxygen supply, carbon to nitrogen ratio, and cycle length [12,14,15]. In general, it has been observed that a very high OLR in selective reactors is related to a decrease in the polyhydroxybutyrate (PHB) storage capacity due to the prevalence of cellular growth [16]. Furthermore, differences on the feeding pattern and substrate concentration have been shown considerable differences in the PHA content and yields [17]. On the other hand, the length of the cycle (total cycle time) has been shown to influence the performance of PHA production as well, increasing the substrate uptake rate when decreasing the cycle length [18].

When considering PHA production using VFA from waste, significant variations in the substrate concentration, composition, and availability may be expected. Changes in conditions will most likely require adjusting operational parameters to ensure efficient selection of an active PHA producing population. This problem could be addressed by adjusting the feast and famine intervals of each operation cycle, according to the influent availability and characteristics. To do so, information is needed about the influence of conditions, such as the feed concentration (which determines OLR) and cycle length of the PHB accumulation. At the same time, different conditions also affect the costs associated with the production of PHA-enriched biomass. Then, conditions favouring maximal PHA accumulation may not coincide with those providing lower costs. The aim of this work is to evaluate the influence of the cycle length and carbon concentration on the dynamics of the feast-famine process. The influence of both parameters was determined using surface response methodology. By means of the kinetics of acetate and oxygen consumption, feast and famine phases were identified and studied. Moreover, costs estimations were performed to evaluate the effect of the same parameters on the first stages of PHA production: Selection, enrichment, and biomass separation. Studying the costs associated with PHA production is relevant, since the price of PHA would be mainly influenced by process costs [19].

## 2. Materials and Methods

### 2.1. Reactor Operation

Several sequential batch bioreactor (SBR) runs were performed. Four SBRs with a working volume of 2 L were implemented for that purpose. All SBRs were operated under ADF conditions, which consisted of a feed period, a variable period of aerobic reaction, and a final withdrawal of the mixed liquor from the mixed vessel. SBRs were operated using acetate as the sole carbon source. The feed and withdrawal periods were 6 and 10 min. The feed flow was 2.5 L/h. Withdraw was set to remove the same volume added during feeding (0.25 L). No settling phase was performed. Then, the sludge retention time (SRT) was equal to the hydraulic retention time (HRT). 

Conditions imposed during SBR runs were organized using surface response methology, applying a face-centred central composite design [20]. Studied factors were the feed concentration and total cycle length (feast + famine). Each factor was studied at 3 levels: 30, 75, and 120 mM and 4, 8, and 12 h for the acetate concentration and total cycle length, respectively. The central point was replicated 3 times. Conditions applied are shown in Table 1 (11 reactor runs). Different responses were studied, such as the feast/famine (F/F) length ratio, biomass productivity, maximum PHB content, and PHB productivity. ANOVA analyses were made for each response. In all cases, the model significance and lack of fit tests were performed to check that models were relevant and fitted the experimental data. All statistical analyses were made considering α = 0.05.

A complementary SBR was run to provide a fairly stable inoculum for each of the reactor runs described in Table 1. This SBR was in turn inoculated with activated sludge from the sewage treatment plant of the city of Temuco (Chile). The feed concentration was 120 mM, and the cycle length was 6 h. During the operation of all SBRs, aeration was provided at an airflow rate of 3–5 L/min. All reactors were also mechanically stirred at 60 rpm. 

The influent was composed of sodium acetate and a mineral medium. The mineral medium composition was 600 mg/L MgSO_4_·7H_2_O, 160 mg/L NH_4_Cl, 100 mg/L mg EDTA, 92 mg/L mg K_2_HPO_4_, 45 mg/L KH_2_PO_4_, 70 mg/L CaCl_2_·2H_2_O, thiourea (10 mg/L), and 2 mL/L of trace elements solution. The trace solution composition was 1500 mg/L FeCl_3_·6H_2_O, 150 mg/L H_3_BO_3_, 150 mg/L CoCl_2_·6H_2_O, 120 mg/L MnCl_2_·4H_2_O, 120 mg/L ZnSO_4_·7H_2_O, 60 mg/L Na_2_MoO_4_·2H_2_O, 30 mg/L CuSO_4_·5H_2_O, and 30 mg/L of KI.

Dissolved oxygen (DO) and pH were acquired online by means of electrodes. DO was measured using an optic industrial probe (WQ401, Global Water, College Station, TX, USA). Signals from sensors and pumps control were handled using a CompactDAQ system (cDAQ-9178 chasis, National Instruments, Austin, TX, USA) and a routine specially programmed for this purpose using LabView software (National Instruments).

### 2.2. Analytical Methods

Acetate was determined by gas-chromatography using a Flame Ionization Detector (Clarus 400, Perkin Elmer), using a Nukol™ capillary column (Sigma-Aldrich, Darmstadt, Germany). The cell dry weight was quantified using the volatile suspended solids (VSS) technique according to Standard Methods (APHA 2011).

PHB determination was performed according to Serafim et al. [21]. 5 mL of homogenized culture were collected, and five drops of formaldehyde were added to stop biological activity: Samples were then frozen and lyophilized for storage. The biomass samples were later resuspended in 1 mL acidic methanol (20% H_2_SO_4_) with 0.65 mg/mL of benzoic acid as the internal standard (Sigma Aldrich™). The chloroform phase was collected and molecular sieves (0.3 nm) were added for water adsorption. One mL of the chloroform phase obtained was injected on-column in the same gas chromatograph used for acetate determination. A calibration curve was prepared by injecting standard concentrations of hydroxybutyric acid sodium salt (Sigma Aldrich) previously submitted to the procedure described for reactor samples.

### 2.3. Estimation of Costs for PHA-enriched Biomass Production

**Figure 1:**
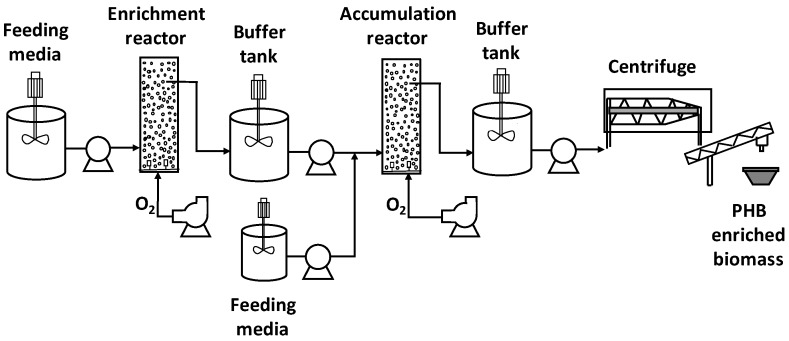
Process considered for costs estimation of biomass-enriched PHB.

An estimation of the costs associated with the production of PHA-enriched biomass was performed, for each of the conditions described in Table 1. Capital and operating costs were considered for the process described in Figure 1. It consisted of three stages: Biomass selection, PHA accumulation, and biomass harvest (by centrifugation). This study included only biomass production (and not PHA extraction or purification) since that stage is the one that will be most affected by any changes of the operational parameters studied in this research. 

Analysis was made considering the following conditions or assumptions:

▪ An annual production of 500 ton of PHA was considered as the calculation basis.
▪ The total volume needed for enrichment and accumulation stages were calculated according to the production rate of PHB of each stage. To calculate the number of reactors required, reactors with a useful volume of 8 m^3^ were considered.
▪ The enrichment reactor produced biomass with a PHA content equal to that observed by the end of each operation cycle tested experimentally. The accumulation reactor produces biomass with a PHA content equal to that observed by the end of each feast cycle tested experimentally.
▪ The pumping capacity needed for each reactor was calculated taking into account the inlet flowrate supplied during the feeding time. Pumping energy consumption was calculated based on the inlet flowrate supplied and considering a reactor height of 4.6 m and an energy efficiency of the pump of 0.7.
▪ The oxygen requirements for the enrichment and accumulation reactors were determined by means of chemical oxygen demand (COD) mass balances. The air flowrate supplied was calculated using a mass transfer efficiency factor of 20%. Fans with a capacity of 3 m^3^ air/s were considered.
▪ The total volume required for the feeding and buffer tanks was determined based on the flowrates of each stream and considering a storage period of 12 h. Tanks with a volume of 60 m^3^ were chosen. Each tank was provided with one agitator whose specific power and energy efficiency were 0.01 kW·h/m^3^ and 0.7, respectively.
▪ To separate the biomass generated during the accumulation stage, centrifuges with a capacity of 30 m^3^/h were considered. A solid separation efficiency of 100% was assumed.
▪ Energy costs were calculated taking into account the energy consumed by agitators, pumps, and fans, considering a price of 15.8 cents USD/kW⋅h.
▪ Capital costs were calculated according to [22]. Prices were updated taking into account an annual increase of costs of 3%. A lifetime of 20 years was considered.

All equations used for costs estimations are reproduced in the Appendix.

## 3. Results and Discussion

### 3.1. SBRs Operation

**Figure 2:**
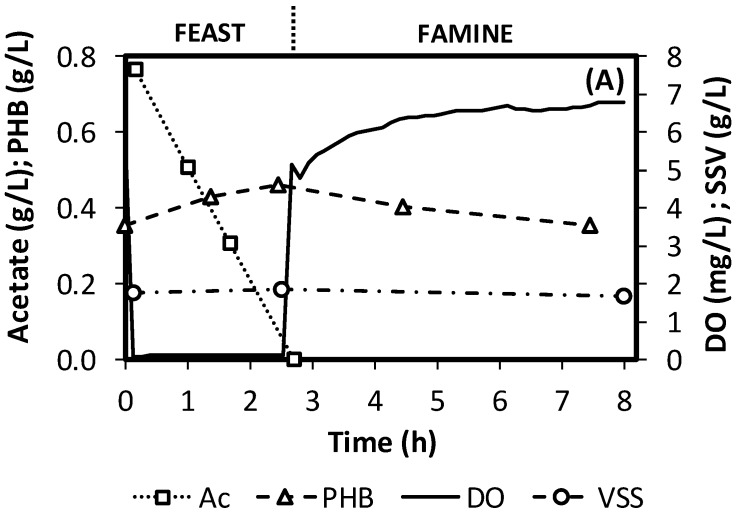

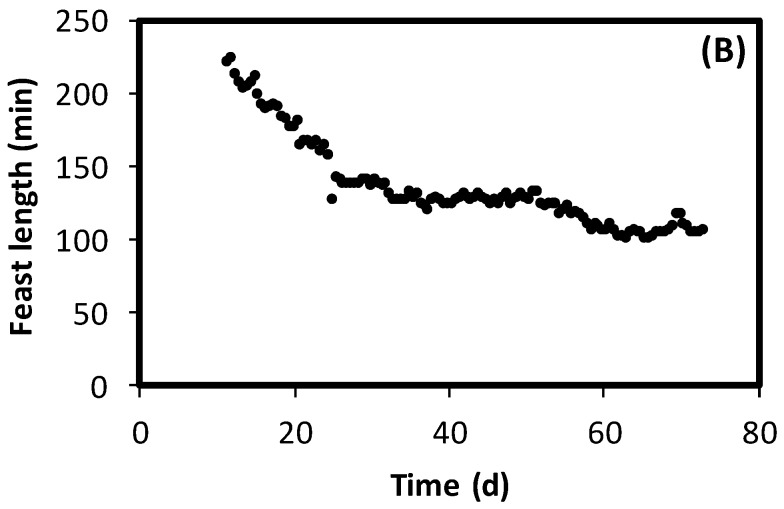
(**A**) Typical behaviour of an operation cycle (SBR #2). (**B**) Evolution of the feast length during operation of SBR #10.

Figure 2A shows a typical operation cycle (data from SBR #2, see Table 1). After feeding, oxygen uptake increases, producing a sudden drop in DO. Oxygen concentration remains almost null, while acetate is present in the reactor, as a result of a high oxygen uptake rate. During this period, biomass PHB content increases. After acetate is depleted, oxygen rapidly increases as the oxygen consumption rate is reduced. As described by previous authors [23], these changes in the patterns of DO correlate well with the limits of the feast and famine phases. Then, the oxygen profile can then easily be used to determine the feast/famine boundary. Figure 2A also shows how PHB is produced during the feast and consumed during the famine. As a result, similar PHB concentrations were observed at the beginning and end of the cycle.

Obviously, the application of the conditions described in Table 1 induced changes in the biomass developed in the reactors. Figure 2B presents the evolution of the feast phase length of SBR #10. It can be observed that the feast length decreases rapidly during the first 30 days of operation. No changes are observed after day 60, indicating that SBR is in a stable state of operation. The time required to reach the steady state depended on the applied operational conditions. To ensure that the responses measured in the design of experiments were representative of the applied conditions, the operation was kept until steady state conditions were achieved, which was identified by a constant feast length. Only then analyses were performed, and responses were evaluated. As a result, all SBRs were operated for a period exceeding 10 SRT. As expected, SBR stabilization took longer in those cases when applied conditions were far from those applied in the SBR operated to provide the inoculum. 

Table 2 presents feast and famine phases lengths for each SBR. Values from the end of the operation are presented, when the stable operation was identified, based on the criteria described above.

### 3.2. Effect of Substrate Concentration and Cycle Length on SBR Operation

**Figure 3:**
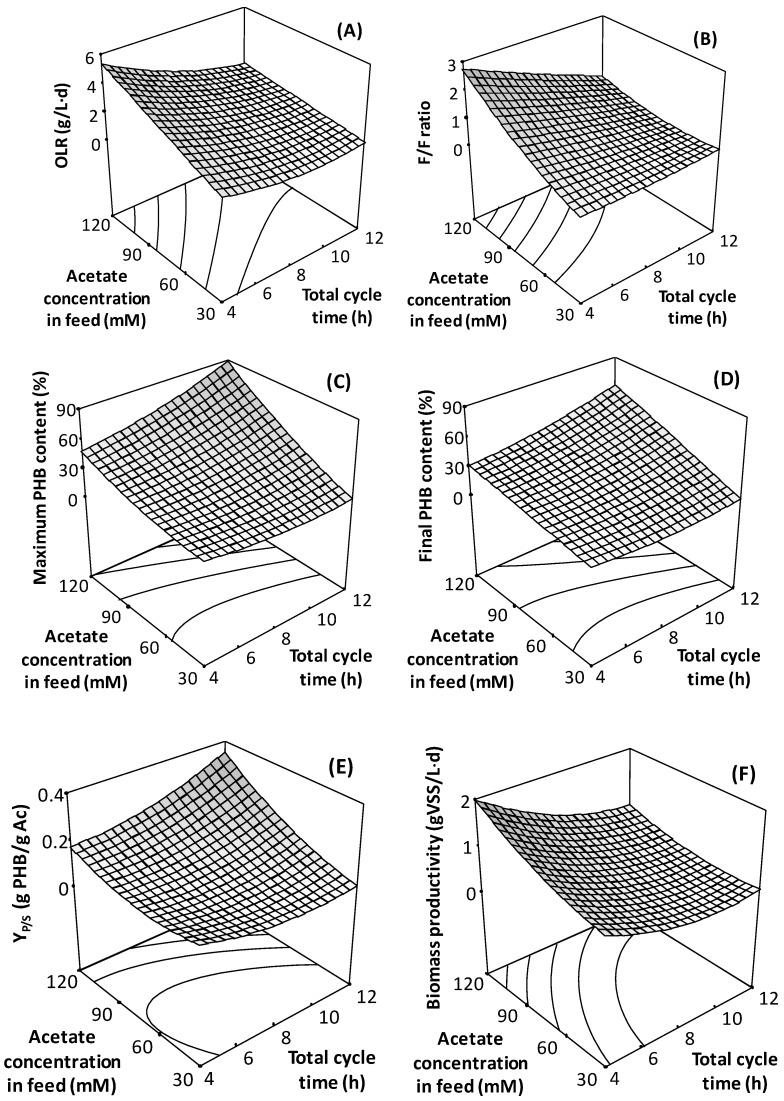
Selected responses observed during surface response methodology. Factors tested: Acetate concentration in the feed and total cycle time. Responses: (**A**) OLR, (**B**) F/F ratio, (**C**) maximum PHB content of biomass, (**D**) PHB content of biomass by the end of the cycle, (**E**) yield PHA/acetic acid, (**F**) biomass productivity.

Figure 3 presents the effect of the studied factors (cycle length and acetate concentration in the feed) over the studied responses. Figure 3A presents the OLR. Since the feed volume per cycle remained constant, changes in the influent concentration resulted in different applied OLRs. On the other hand, increases in the cycle time reduced the applied loading. As a result, OLRs were in the range of 0.5–5.5 g acetic acid/L·d. Figure 3B presents the F/F ratios, which is the relation between times presented in Table 2. Figure shows a direct relation between the F/F ratio and acetate concentration, and an inverse relation with the cycle length. Such a result is expected, since a higher feed concentration results in a higher loading rate, requiring more time for the culture to consume substrate, increasing the feast time. On the other hand, a higher total cycle will result in a higher famine time. In general, the F/F ratios observed in this study are high when compared with the ones reported by other authors [24,25,26], who normally applied values below 0.5. Famine lengths observed in this study were in the range of 1–11 h (see Table 2). 

Figure 3C presents the maximum biomass PHB content, which is attained by the end of the feast period (beginning of famine), as can be seen in Figure 2A. The maximum PHB content ranged between 10% and 90%, revealing how determinant the studied parameters are in terms of selecting microorganisms with a high PHB storage capacity. An increase in the cycle time induced an increment on the maximum PHB content. This is most likely the result of the consequent increment of the famine length. Under tested conditions, the feast is mainly a function of the applied acetate concentration, so increases of the cycle time at a constant acetate concentration will increase the famine length. Decreasing F/F enhances growth limitation, favouring PHA storage [24]. On the other hand, a clear increase in the maximum PHB content was observed when increasing the acetate concentration (and, therefore, the organic loading rate), behaviour that has already been already observed in previous studies [16,27].

**Figure 4:**
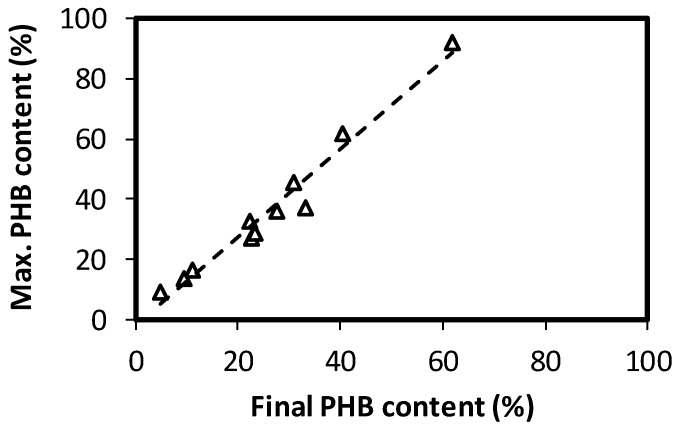
Relation between the maximum and final PHB contents for the operation of SBR reactors.

Considering that the draw of biomass takes place by the end of each cycle, the final PHB content, i.e., the one by the end of famine, is probably a more relevant information for reactor operation, than maximum content (see Figure 3D). As expected, values are lower than those shown in Figure 3C, because of PHB consumption during the famine. Nevertheless, a constant relation between the maximum and final PHB contents was observed, irrespective of the conditions tested, as can be seen in Figure 4. It is clear that during famine, about 30% of the PHB was consumed. Proportionality between the maximum and final PHB content indicates that the second value can be a good indicator of the first one. Then, decisions may be taken based on the PHB content at the end of the cycle, which may be easier to determine under full-scale operational conditions, or when detailed control or follow up of the reactor may not be feasible. 

A similar trend as that described for the biomass PHB content can be observed for Y_P/S_, the product/substrate yield (Figure 3E). This value has been computed considering the maximum PHB biomass content. As expected, conditions providing a higher Y_P/S_ are those generating a higher PHB biomass content, since more substrate is oriented towards polymers’ accumulation, as already described. 

Results correspond to the selection reactors, which will produce biomass for an accumulation step, where further conditions may be imposed to enhance PHB accumulation (such as nitrogen limitation). Then, biomass productivity is also relevant for full-scale application, since a higher productivity will produce more biomass to potentially feed such an accumulating reactor. Biomass productivity, in terms of VSS, is presented in Figure 3F. The average productivity is presented, i.e., the mass of biomass exiting the reactor after each cycle per volume of reactor divided by the cycle length. Values presented correspond to the total biomass, including the intracellular accumulated PHB. As expected, biomass productivity follows the same pattern as the applied OLR (Figure 3A), since complete substrate consumption is obtained under all conditions. Comparison of Figure 3D,F shows a compromise between the biomass production and observed PHA accumulation, a phenomenon that has been previously descried [16,23]. Depending on the eventual performance of an accumulation step, a lower content of PHB may be accepted if a higher amount of biomass can be produced in the selection step. A parameter that may provide useful criteria for the determination of operational conditions could be PHB productivity during selection reactors operation. Unfortunately, PHB productivity cannot be presented in the form of a surface response, since ANOVA analysis showed a significant lack of fit (α = 5%), meaning that the second order model used does not correctly represent the observed response variation. Then, the observed PHB productivity is presented in Table 3. Two productivities can be evaluated, one in terms of the maximum PHB content (observed by the end of the feast) and one based on the amount of PHB at the end of the cycle. The first productivity would be the one observed if biomass is harvested by the end of the feast. Both productivities are proportional, as is the case of both biomass PHB contents (Figure 4). 

### 3.3. Estimation of Costs for PHA-Enriched Biomass Production

**Figure 5:**
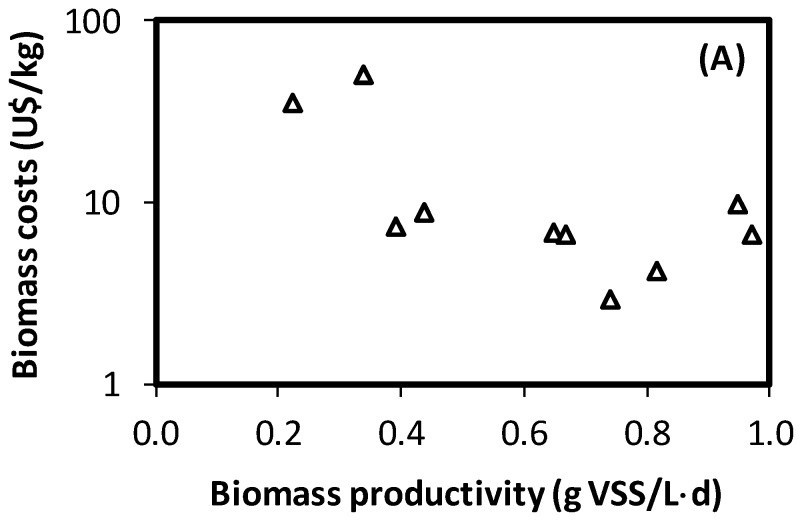

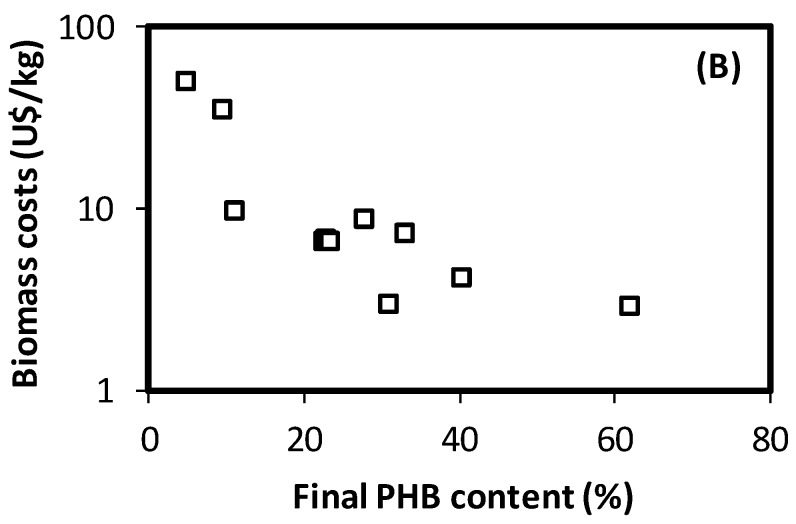
Cost estimation for production of PHA-enriched biomass as a function of (**A**) the total biomass productivity and (**B**) PHB content by the end of the cycle.

Figure 5 shows the costs estimation for PHA-enriched biomass, as a function of the biomass productivity and PHA content by the end of each cycle. To construct these charts, the data coming from the experiments described in Table 1 were used. Few reports are available dealing with costs of PHA production in literature, such as [28,29]. These studies are very useful to determine the industrial applicability of mixed culture based PHB production. However, the results are greatly dependent on the particular conditions and process selected for the analyses. In the case of this study, a sole reactor volume was considered, so the replication of reactors was considered when higher fermentation volumes were required. Then, the economy of scale is not taken into consideration. However, it provides an opportunity to visualise the potential impact of operational conditions on potential associated costs. Observation of Figure 5A,B clearly shows the already identified compromise between biomass productivity and PHA content. Conditions providing a high level of PHB accumulation in the selection process involve low biomass productivity, which in turn affect overall costs. Conditions tested in this research provided a wide range of PHB contents and biomass productivities, producing great differences in the associated costs for biomass production. Results provided by this research confirms the great relevance that selection appropriate conditions for biomass selection can produce on PHB-enriched biomass quality and production costs. Results also suggests that PHB content alone may not be a reasonable criterion for determining optimal conditions for PHB production. If costs need to be reduced, conditions providing a lower PHB content in the selection reactor, but a higher biomass productivity may be of interest, as long as the reduction of PHB content does not involve significant increases in PHB extraction and purification costs.

## 4. Conclusions

Substrate concentration and cycle length proved to have a deep impact on SBR operation for the selection of PHB-accumulating biomass. Both factors were shown to have statistically significant effects over biomass productivity, PHB content, and product/substrate yield. The PHB content by the end of the feast and famine stages was shown to have a constant relation, irrespective of the conditions tested. In all cases, 30% of the existing PHB was consumed during famine. Biomass productivity was expected to have a relevant effect on the costs associated with the production of PHA-enriched biomass. Since the results showed a negative relation between biomass productivity and PHA content, costs for biomass production are higher for those conditions providing higher PHA contents in the biomass. Thus, maximising PHB content in the selection reactor may provide excessive costs for biomass production. 

## Figures and Tables

**Table 1:** Operational conditions in all enrichment SBRs from the experimental design.

Run	Cycle Length (h)	Acetate Concentration in the Feed (mM)	Organic Loading Rate (g/L·d)	SRT * (d)
SBR #1	4	30	1.4	1.3
SBR #2	4	75	3.4	1.3
SBR #3	4	120	5.4	1.3
SBR #4	8	30	0.7	2.7
SBR #5	8	75	1.7	2.7
SBR #6	8	75	1.7	2.7
SBR #7	8	75	1.7	2.7
SBR #8	8	120	2.7	2.7
SBR #9	12	30	0.5	4.0
SBR #10	12	75	1.1	4.0
SBR #11	12	120	1.8	4.0

* Since no biomass retention was applied, SRT is equal to HRT.

**Table 2:** Feast and famine phases length (by the end of operation) for the SBR runs described in Table 1.

Run	Feast Length (h)	Famine Length (h)
SBR #1	0.88	3.12
SBR #2	1.90	2.10
SBR #3	2.97	1.03
SBR #4	0.81	7.19
SBR #5	2.92	5.08
SBR #6	3.40	4.60
SBR #7	2.86	5.14
SBR #8	4.79	3.21
SBR #9	1.15	10.85
SBR #10	2.85	9.15
SBR #11	4.38	7.63

**Table 3:** PHB average productivity for the operation of SBR.

Run	PHB Productivity Based on Maximum PHB (g/L·d)	PHB Productivity Based on Final PHB (g/L·d)
SBR #1	0.16	0.10
SBR #2	0.32	0.22
SBR #3	1.01	0.68
SBR #4	0.03	0.02
SBR #5	0.18	0.15
SBR #6	0.19	0.16
SBR #7	0.16	0.12
SBR #8	0.51	0.33
SBR #9	0.03	0.02
SBR #10	0.15	0.13
SBR #11	0.68	0.46

## Appendix: Equations for Costs Estimation of PHA-Enriched Biomass

Capital and operating costs were calculated for an annual production of PHB of 500 Tons.

Number of SBRs for PHB accumulation:

$$N_{\;SBR\;Accum}=round.up(\frac{PHB\;production(\frac{Ton\;PHB}{year})\cdot 1000(\frac{kg\;PHB}{Ton\;PHB})\cdot \frac{1}{365}(\frac{year}{d})}{\left[{\left(r_{PHB}\right)}_{feast}\cdot (\frac{kg\;PHB}{m^{3}\cdot d})\cdot \frac{t_{feast}}{t_{fed}+t_{feast}+t_{withdrawal}}+{\left[VSS\right]}_{end\;of\;enrichment\;cycle}(\frac{kg\;VSS}{m^{3}})\cdot {PHH}_{fraction\;end\;of\;enrichment\;cycle}(\frac{kg\;PHB}{kg\;VSS})\cdot (1-{VF}_{fed}\cdot \frac{24\;(\frac{h}{d})}{\left(t_{fed}+t_{feast}+t_{withdrawal})\;(h\right)}\right]\cdot V_{r}(\frac{m^{3}}{reactor})})+1$$

VF_fed_= Volumetric fraction of feeding media supplied per cycle (1/8); V_r_= Useful volume of the reactor (8.0 m^3^; Total volume: 10.5 m^3^; H/D: 4)

Number of SBRs needed to carry out biomass enrichment:

$$N_{\;SBR\;Enrichment}=round.up(\frac{PHB\;production(\frac{Ton\;PHB}{year})\cdot 1000(\frac{kg\;PHB}{Ton\;PHB})\cdot \frac{1}{365}(\frac{year}{d})-{\left(r_{PHB}\right)}_{feast}\cdot (\frac{kg\;PHB}{m^{3}\cdot d})\cdot \frac{t_{feast}}{t_{fed}+t_{feast}+t_{withdrawal}}\cdot ({N_{SBR}}_{Accum}-1)\;\left(reactor\right)\cdot V_{r}\;(\frac{m^{3}}{reactor})}{\left[{\left[VSS\right]}_{end\;of\;enrichment\;cycle}(\frac{kg\;VSS}{m^{3}})\cdot {PHH}_{fraction\;end\;of\;enrichment\;cycle}(\frac{kg\;PHB}{kg\;VSS})\cdot \frac{24\;(\frac{h}{d})}{tcycle\;Enrichment\;(h)}]\cdot V_{r}(\frac{m^{3}}{reactor}\right)})+1$$

VER_Enrichment_= Volumetric exchange ratio of the enrichment reactor (1/8); V_r_= Useful volume of the reactor (8.0 m^3^; Total volume: 10.5 m^3^; H/D: 4)

Flow rate fed of SBRs for PHB accumulation:

$$Flowrate_{Fed\;Acum}\;\left(\frac{m^{3}}{d}\right)=VF_{fed}\;\cdot V_{r}\;\left(\frac{m^{3}}{reactor}\right)\cdot (N_{SBR_{Accum}}-1)\;\left(reactor\right)\cdot \frac{24\;\left(\frac{h}{d}\right)}{(t_{fed}+t_{feast}+t_{withdrawal})\;\left(h\right)}$$

Flow rate from enrichment SBRs to SBRs for PHB accumulation:

$$Flowrate_{Enrich\;Accum}\;\left(\frac{m^{3}}{d}\right)=(1-VF_{fed})\;\cdot V_{r}\;\left(\frac{m^{3}}{reactor}\right)\cdot (N_{SBR_{Accum}}-1)\;\left(reactor\right)\cdot \frac{24\;\left(\frac{h}{d}\right)}{(t_{fed}+t_{feast}+t_{withdrawal})\;\left(h\right)}$$

Flow rate fed of SBRs for PHB accumulation:

$$Flowrate_{Fed\;Enrich}\;\left(\frac{m^{3}}{d}\right)=VER_{Enrichment}\;\cdot V_{r}\;\left(\frac{m^{3}}{reactor}\right)\cdot (N_{SBR_{Enrich}}-1)\;\left(reactor\right)\cdot \frac{24\;\left(\frac{h}{d}\right)}{t_{cycle\;Enrichment}\;\left(h\right)}$$

Pumping capacity:

$$Flowrate\;Pump_{Fed\;Accum}\;\left(\frac{m^{3}}{s}\right)=\frac{VF_{fed}\;\cdot V_{r\;Accum}\left(m^{3}\right)}{t_{fed}\left(\min\right)\cdot \frac{1}{60}\left(\frac{min}{s}\right)}$$

$$Flowrate\;Pump_{Enrich-Accum}\;\left(\frac{m^{3}}{s}\right)=\frac{\left(1-VF_{fed}\right)\cdot V_{r\;Accum}\left(m^{3}\right)}{t_{fed}\left(\min\right)\cdot \frac{1}{60}\left(\frac{min}{s}\right)}$$

$$Flowrate\;Pump_{Fed\;Enrichment}\;\left(\frac{m^{3}}{s}\right)=\frac{VER_{Enrichment}\;\cdot V_{r\;Enrichment}\left(m^{3}\right)}{t_{fed}\left(\min\right)\cdot \frac{1}{60}\left(\frac{min}{s}\right)}$$

Number of pumps:

$$N_{Pump\;fed\;Accum\;}=round.up\;\left(\frac{Flowrate_{Fed\;Accum}\;\left(\frac{m^{3}}{d}\right)}{Flowrate\;Pump_{Fed\;Accum}\;\left(\frac{m^{3}}{s}\right)\cdot 86400\;\left(\frac{s}{d}\right)}\right)+1$$

$$N_{Pump\;Enrich-Accum\;}=round.up\;\left(\frac{Flowrate_{Enrich-Accum}\;\left(\frac{m^{3}}{d}\right)}{Flowrate\;Pump_{Enrich-Accum}\;\left(\frac{m^{3}}{s}\right)\cdot 86400\;\left(\frac{s}{d}\right)}\right)+1$$

$$N_{Pump\;fed\;Enrichment\;}=round.up\;\left(\frac{Flowrate_{Fed\;Enrich}\;\left(\frac{m^{3}}{d}\right)}{Flowrate\;Pump_{Fed\;Enrichment}\;\left(\frac{m^{3}}{s}\right)\cdot 86400\;\left(\frac{s}{d}\right)}\right)+1$$

Pumping energy consumption:

$$Pump\;Power\;\left(W\right)=\frac{\rho_{H_{2}O\;\left(\frac{kg}{m^{3}}\right)\cdot Flowrate\;Pump\;\left(\frac{m^{3}}{s}\right)\cdot H_{r}\left(m\right)}}{\eta_{pump}}$$

η_pump_= Energy efficiency of the pump (0.7); H_r_= height of reactor (4.6 m)

$$Pumping\;energy\;consumption_{\;Fed\;Accum}\left(\frac{kW\cdot h}{d}\right)=\frac{Pump\;Power_{Fed\;Accum}\;\left(W\right)\cdot Flowrate_{Fed\;Accum}\;\left(\frac{m^{3}}{d}\right)}{Flowrate\;Pump_{Fed\;Accum}\;\left(\frac{m^{3}}{s}\right)\cdot 3.6\cdot 10^{6}\;\left(W/\left(\frac{kW\cdot h}{d}\right)\right)}$$

Sodium acetate consumption:

$$Inlet\;AcNa_{Enrichment}\;\left(\frac{kg\;AcNa}{d}\right)=\;{\left[AcNa\right]}_{fed}\left(\frac{kg\;AcNa}{m^{3}}\right)\cdot Flowrate_{Fed\;Enrichment}\;\left(\frac{m^{3}}{d}\right)$$

$$Inlet\;AcNa_{Accum}\;\left(\frac{kg\;AcNa}{d}\right)=\;{\left[AcNa\right]}_{fed}\left(\frac{kg\;AcNa}{m^{3}}\right)\cdot Flowrate_{Fed\;Acum}\;\left(\frac{m^{3}}{d}\right).\;$$

Oxygen consumption: Oxygen consumed during the operation of both the enrichment and accumulation systems was calculated based on a COD balance:

$$m_{O_{2}\;}\left(\frac{kg\;O_{2}}{d}\right)=\;m_{AcNa_{i}}\left(\frac{kg\;AcNa}{d}\right)\cdot \left[0.78\;\left(\frac{kg\;COD}{kg\;AcNa}\right)-Y_{\frac{PHB}{AcNa}}\left(\frac{kg\;PHB}{kg\;AcNa}\right)\cdot 1,67\left(\frac{kg\;COD}{kg\;PHB}\right)-Y_{\frac{X}{AcNa}}\left(\frac{kg\;Biomass}{kg\;AcNa}\right)\cdot 1,42\left(\frac{kg\;COD}{kg\;Biomass}\right)\;\right]$$

Fan flow rate calculation:

$$Q_{O_{2}}\left(\frac{m^{3}{}_{air}}{s}\right)=\frac{m_{O_{2}\;}\left(\frac{kg\;O_{2}}{d}\right)\cdot 22.4\left(\frac{m^{3}{}_{O_{2}}}{kmol\;O_{2}}\right)}{32\;\left(\frac{kg\;O_{2}}{kmol\;O{\;}_{2}}\right)\cdot 0.21\;\left(\frac{m^{3}{}_{O_{2}}}{m^{3}{}_{air}}\right)\cdot 86400\;\left(\frac{s}{d}\right)\cdot 0.2\left(\frac{kg\;O_{2}transfered}{kg\;O_{2}supplied}\right)}$$

Number of fan:

$$Number\;of\;fan_{Accum}=round.up\left(\frac{Q_{O_{2}}\left(\frac{m^{3}{}_{air}}{s}\right)}{Fan\;Flowrate\;\left(\frac{m^{3}{}_{air}}{s\cdot fan}\right)\cdot \frac{t_{feast}}{t_{fed}+t_{feast}+t_{withdrawal}}}\right)+1$$

$$Number\;of\;fan_{Enrichment}=round.up\left(\frac{Q_{O_{2}}\left(\frac{m^{3}{}_{air}}{s}\right)}{Fan\;Flowrate\;\left(\frac{m^{3}{}_{air}}{s\cdot fan}\right)\cdot \frac{t_{feast}+t_{famine}}{t_{fed}+t_{feast}+t_{famine}+t_{withdrawal}}}\right)+1$$

Fan flowrate: 3 m^3^ air/s

$$Fan\;energy\;consumption\;\left(\frac{kW\cdot h}{d}\right)=1\;\frac{kW\cdot h}{kg\;O_{2}\;}\cdot \left(m_{O_{2}Enrich\;}+m_{O_{2}\;Acum}\right)\left(\frac{kg\;O_{2}}{d}\right)$$

Storage and buffer tanks:

To determine the total volume of tanks required in each stage, a storage period of 12 h was considered.

$$Number\;of\;storage\;tanks=round.up\left(\frac{Flowrate\;\left(\frac{m^{3}}{d}\right)\cdot 0.5\;d}{Volume\;of\;tank\;\left(m^{3}\right)}\right)+1$$

Volume of tank: 60 m^3^

Agitators:

One agitator per tank was selected and the required power was calculated considering 0.01 kW⋅h/m^3^ and an energy efficiency of 0.7.

Solids generated:

$$VSSgenerated\;\left(\frac{kg\;VSS}{d}\right)=\;\frac{PHB\;production\;\left(\frac{Ton\;PHB}{year}\right)\cdot 1000\left(\frac{kg\;PHB}{Ton\;PHB}\right)\cdot \frac{1}{365}\left(\frac{year}{d}\right)}{PHB_{fraction\;feast}\left(\frac{\;kg\;PHB}{kg\;VSS}\right)}$$

Centrifuge energy consumption:

$$Centrifuge\;energy\;consumption\;\left(\frac{kW\cdot h}{d}\right)=0.3\;\frac{kW\cdot h}{kg\;VSS\;}\cdot VSSgenerated\;\left(\frac{kg\;VSS}{d}\right)$$

Number of centrifuges (centrifuge capacity: 30 m^3^/h):

$$N_{centrifuge}=round.up\left(\frac{(Flowrate_{Enrichment-Accum}+Flowrate_{Fed-Accum})\left(\frac{m^{3}}{d}\right)\cdot \frac{1}{24}\left(\frac{h}{d}\right)}{Centrifuge\;capacity\;\left(\frac{m^{3}}{h}\right)}\right)+1$$
